# Quantifying and Minimizing the Variance of Gradient Insulator-Based Dielectrophoresis

**DOI:** 10.3390/mi17050600

**Published:** 2026-05-14

**Authors:** Hoai Nguyen, A. K. M. Fazlul Karim Rasel, Mark A. Hayes

**Affiliations:** School of Molecular Sciences, Arizona State University, Tempe, AZ 85287, USA; hoainguyen@asu.edu (H.N.); arasel@asu.edu (A.K.M.F.K.R.)

**Keywords:** dielectrophoresis, electrophoresis, electrokinetic, microfluidic, particle dynamics, finite numerical simulation

## Abstract

Opportunities abound in microfluidic technologies to impact how we understand extremely complex systems with many constituents which change with time and space. In these technologies, separation science plays a central role towards understanding everything from biology and healthcare to environmental monitoring to the search for life in the Solar system. Separations can amplify the capabilities of detection modalities by isolating targets and/or increasing their concentration while removing background constituents which can interfere with their sensing. In essence, separations increase the amount of information that can be gathered from a sample. The ideal features of next-generation separations capability are present in gradient insulator-based dielectrophoresis (g-iDEP), enabled by the length scale and precision of microfluidics. It acts through electric field interactions with particles, which enables unbiased (label-free) separations since all relevant particles, from atoms to cells, have an accessible response to electricity—either through linear (electrophoresis) or higher-order gradient (dielectrophoresis and related) effects. The technique isolates and concentrates, enabling improved detection function and multidimensional separations. Its foundational theoretical capabilities give it separations power on the order of 1:10^8^, beyond the resolving power of the best mass spectrometers and ultra-high resolution spectroscopies. Experimental evidence is amassing that shows it to be a powerful tool that can resolve tiny differences in cells (antibiotic resistance versus susceptible in unlabeled paired isolates across many species) and differentiate single-point mutations in proteins. Its capabilities are still emerging, and this work aims to quantify the current practice and connect those approaches to the ultimate capabilities of the technique towards quantifying the dynamic range and resolving power of the strategy as a whole. The technique uses two methods of quantifying the electrophysical properties of the target, voltage sweep and spatial methods. The voltage sweep method is lower-resolution and serves as a search mode, while the spatial method is higher-resolution and quantifies the properties over a smaller defined range determined via the sweep method. These quantification methods are examined by collating existing experimental data, performing relevant Monte Carlo simulations, and finite element model calculations. These are summarized to understand the mechanisms currently limiting the technique, facilitate quantitative comparisons with traditional separation science capabilities in terms of resolution and dynamic range, and compare them to the theoretical limits of the strategy.

## 1. Introduction

The analysis of particle dynamics within microfluidic systems has become pivotal in advancing both diagnostics and fundamental research. A key unresolved challenge, and the focus of this work, is quantifying the variance that governs the resolution limits of these separations, particularly when distinguishing closely related biological populations. The precise manipulation of particles through electric fields, such as dielectrophoresis (DEP) and electrophoresis (EP), offers unparalleled control in separating and characterizing particles based on their physical properties [1], including but not limited to size [2], charge [3,4] and impedance [5,6]. Significant theoretical and practical advances in gradient-based separations of bioparticles ranging from proteins to cells have been demonstrated using gradient insulator-based dielectrophoresis (g-iDEP) [7,8]. These studies have shown data consistent with theoretical models, which predict one part in 100 million resolving power for any electrostatic or electrodynamic difference in bio-particles and molecules, a difference in particles and bioparticles as small as 1:10^4^ for diameter (m), 1:10^8^ for dielectrophoretic parameters mobility (m^4^V^−2^s^−1^), and 1:10^5^ for electrophoretic mobility (m^2^V^−1^s^−1^) [9]. Despite this extraordinary theoretical resolving power, the practical limits imposed by microchannel design and process variance remain poorly defined.

**Figure 1:**
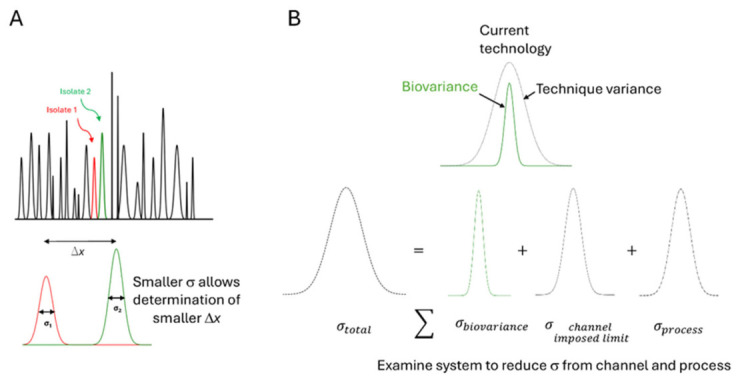
Visualization of biophysical measurements for differentiating bacterial strains. (**A**) The sum of all variances $\sum \sigma_{biovariance}^{2}+\;\sigma_{channel\;imposed\;limit}^{2}+\sigma_{process}^{2}=\sigma_{total}^{2}$ is the observed variance from a measurement. Each strain has a specific biophysical property resulting in a specific value of *x*, for which any closest pair of values is Δx. For strains to be differentiated using biophysical measurements, an accepted metric Δx must be larger than 4σ (σ: standard deviation; full width at half maximum = 2.35σ) total (less than 2.3% overlap of measured populations). (**B**) Any measurement of a biological system includes natural variance ($\sigma_{biovariance}^{2}$) within a population that does not change its clinical or taxonomic assignment. That measurement also includes a variance reflecting any limitations imposed by the instrument ($\sigma_{channel\;imposed\;limit}^{2}$, for gradient insulator-based dielectrophoresis) and processing ($\sigma_{process}^{2}$) of the sample. Existing experimental data for closely related strains, as characterized by genotype or phenotype, show that Δx is larger than 4σ total for all queries using current biophysical differentiation technology (see text). This work examines the sources and magnitude of variance from the processes and instruments used during the measurement enabling an orders-of-magnitude estimation of the $\sigma_{biovariance}^{2}$ from existing data. This will help define the ultimate limits for the determination of $\sigma_{total}^{2}$ and therefore the smallest Δx that may be quantitatively determined. Biophysical characterization is orthogonal to and complimentary to genotyping and phenotyping: this work will better inform the relationships between these cell characterization systems.

A significant driver for this study is the quantification of natural biological variance to study biological heterogeneity. In biological systems where a population is not homogeneous, i.e., a subpopulation of clinically significant bioparticles, such as hetero-resistant pathogenic bacteria cells [10], tumor-derived vesicles [11] or stem cell-derived exosomes [12], variance can provide critical information about subpopulation structure [13,14], disease progression [15,16], or treatment response [17,18]. However, the reliable detection of such closely related bioparticles requires resolving subtle differences in electrokinetic response. Without thorough quantification of this variance, valuable diagnostic information may be lost within unresolved or overlapping populations [19]. The feasibility of resolving closely related populations relies on three elements: how different are the two closest strains or isolates on the axis of differentiation (summed biophysical properties, Δx), “how broad the peaks are” i.e., the variance of each strain or isolate ($\sigma_{x}$^2^) (Figure 1) and the total range of values available for inquiry (dynamic range). While studies have shown that the onset voltage is a function of particle properties and electric field strength [9,20], the ability to predict the resolution of particle populations remains uncertain. A key challenge lies in reconciling onset voltage with population variance, particularly when resolving particles that differ only slightly in mobility.

This paper seeks to address these gaps by exploring the interplay between variance, onset voltage, and the physical constraints imposed by microchannel design. Experimentally, g-iDEP separation can be achieved through two complementary approaches: (1) voltage sweep, where linear regression of particle capture versus applied voltage establishes the onset voltage for dielectrophoretic trapping [21,22] and (2) spatial separation, where particles with different dielectrophoretic properties are trapped at distinct positions along the channel center line [23]. By examining a range of (bio)particles and voltage regimes in triangular-insulator gradient dielectrophoresis microdevices [24,25,26], we aim to provide a comprehensive framework for understanding how these factors influence the resolution and dynamic range of particle separations in DEP systems. The focus is on pathogenic bacterial cells; however, the approach can be adapted to a range of bioparticles.

## 2. Materials and Methods

### 2.1. Estimating the Variance of the Voltage Sweep Method in Determination of the Onset Voltage

A common method of estimating the onset voltage [21,22,27,28], hence deriving the electrokinetic mobility ratio (EKMr), is based on finding the baseline intercept of the linear regression between *x* (voltage) and *y* (the number or intensity (*I*)) of captured bioparticles (*N*). The linear regression provides an estimate of the best fit line for a set of *x* − *y* scatter plot data resulting in a simple linear relationship, y=ax+b and onset voltage is $\frac{y-b}{a}$. Consider a uniform population of particles moving with an average velocity v in a microchannel. Over an interval of time t, each particle travels a distance d=vt. The number of particles N that arrive at the constriction zone is:

(1) N=n ×v ×t×w×h

where n is particle density, and the dimension of the microchannel can be expressed in h, the height of the channel, and width w, the width of the channel. Velocity v can be represented as the product of the electric field and electrokinetic mobility $\mu_{EK}$ (which includes both electroosmosis and electrophoresis), and Equation (1) can be rewritten as:

(2) $$N=n\;\times \mu_{EK}\times E_{ave}\;\times t\times w\times h\;,$$

The electric field $E_{ave}$ is the mean field magnitude distributed across the full length of the channel and reservoir, while the local electric field magnitude (E) fluctuates considerably throughout the microchannel due to the influence of insulating features. The local maxima of the electric field magnitude ($E_{max}$) is associated with the location of capture. The applied potential, which is the voltage imposed across the entire microchannel, serves as a practical reference point for expressing $E_{ave}$ and $E_{max}$ in terms of a single, externally controlled parameter. Hence, $E_{ave}$ can be expressed in resistance $R_{ave}$, current $i_{ave}$ and length *L* of the microchannel. Equation (2) becomes:

(3) $$N\;=n\;\times \mu_{EK}\times \frac{R_{ave}\;\times i_{ave}}{L}\;\times t\times \;w\times h\;,$$

where i ×t is the charge passing through the microchannel. Hence, Equation (3) can also be written as:

(4) $$N\;=n\;\times \mu_{EK}\times R\;\times Q\times \frac{w\times h\;}{L}\;,$$

where Q the flow of charge passing through the microchannel in the interval time t. Error of the slope in a linear regression is commonly expressed as:

(5) $$s=\sqrt{\frac{1}{n-2}\frac{\sum {\left(y-y_{i}\right)}^{2}}{\sum {\left(x-x_{i}\right)}^{2}}}.$$

Error propagation of *N*, as formalized in (4) from its individual contribution, assuming that the dimension of the microchannel $\frac{w\times h\;}{L}$ is kept constant, can be written as:

(6) $$\sigma_{N}=N\times \sqrt{{\left[\frac{\sigma_{n}}{n}\right]}^{2}+{\left[\frac{\sigma_{\mu_{EK}}}{\mu_{EK}}\right]}^{2}+{\left[\frac{\sigma_{R\left(\gamma \right)}}{R\left(\gamma \right)}\right]}^{2}+{\left[\frac{\sigma_{Q}}{Q}\right]}^{2}}$$

Combining Equation (5) and (6), assuming that $\sum {\left(y-y_{i}\right)}^{2}$ = $\sigma_{N}$ if the relationship is linear, then:

(7) $$s=\chi \sqrt{\frac{1}{n-2}\frac{N\times \sqrt{{\left[\frac{\sigma_{n}}{n}\right]}^{2}+{\left[\frac{\sigma_{\mu_{EK}}}{\mu_{EK}}\right]}^{2}+{\left[\frac{\sigma_{R\left(\gamma \right)}}{R\left(\gamma \right)}\right]}^{2}+{\left[\frac{\sigma_{Q}}{Q}\right]}^{2}}}{\sum {\left(x-x_{i}\right)}^{2}}},$$

where χ is the correction factor to account for experimental errors.

The measurement error in voltage is small (see Figure S1); therefore, uncertainty in the estimated slope is dominated by variability in the number of bacteria captured (*N*) rather than voltage measurement.

Each term in Equation (7) was estimated from experimental data, as detailed in Supplementary Materials. A Monte Carlo simulation, using Python (version 3.9.6) and NumPy (version 1.21.2) was used for numerical computations. In each of total 10,000 iterations, values for *N* and all variance components were sampled from uniform distributions spanning their experimentally observed ranges. $\mu_{EK}$, $R\left(\gamma \right)$, and Q were measured independently and a reasonable range of values can be found summarized in Table S2. The measurement uncertainties of those are correlated through zeta potential and ionic strength of the buffer [29,30]. Assuming moderate correlation, correlation coefficients ≈ ±0.5 were incorporated into the simulation with multivariate sampling (Figure S4). Bacteria density (n) was treated as independent. This approach allows us to propagate uncertainties from multiple experimental sources through our linear regression analysis, providing a more accurate estimate of the true uncertainty in our slope determination than traditional analytical error propagation methods. This is detailed in Supplementary Materials.

With the estimated slope, the onset voltage was then determined as (*y* − *b*)/*a*. Using sample data of a spherical bacterial isolate (*Staphylococcus epidermidis* ATCC 35984), a distribution of onset voltage was determined. Gaussian noise at three different levels (0%, 50% and 100%) was added to the recorded bacteria number (*y*) to bracket possible variability of onset voltage. The data to calculate the onset voltage range and full code can be found in Supplementary Materials.

### 2.2. Finite Element Modeling to Define Spatial Determination and Inform Voltage Sweep Method

**Figure 2:**
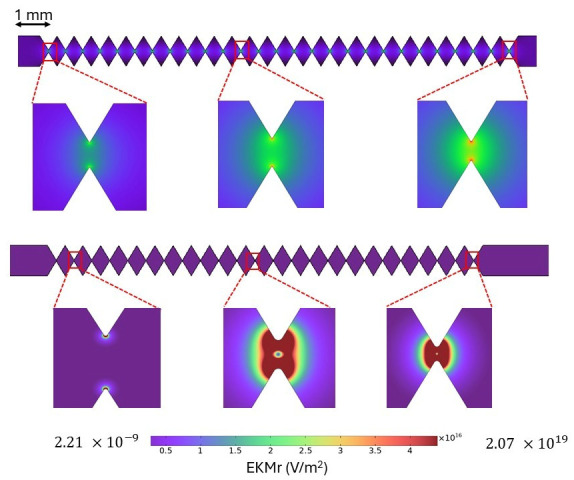
Microchannel designs for numerical modeling of V2L (**top**) and V2S (**bottom**) for generating nonlinear electric field gradients in g-iDEP. Each design comprises threegate sets with progressively decreasing widths from inlet (**left**) to outlet (**right**). The V2L device contains 27 gates with a minimum gap of 27 μm, whereas V2S features 24 gates and a minimum gap of 3 μm. The heat map depicts the electric field magnitude, with warmer colors indicating higher field intensity. Finite element modeling results were focused on the terminal three-gate set for each design, where the field and local gradient are at their maximum values.

Finite element numerical methods are common to predict the fluid flow, the electric field, and gradient of the electric field for DEP microfluidic architectures [31,32]. A two-dimensional simulation based on finite element analysis was performed to mimic the electrokinetic behavior of *S. epidermidis* in a microfluidic device (Figure 2). The simulation is considered in two dimensions to make the simulation computationally cost-effective. For easier calculation, the reservoirs at two opposing ends are omitted. The geometry of the device is drawn in Autodesk AutoCAD 2022. The device design is imported into COMSOL Multiphysics 6.1. A buffer material is used to fill the microchannel. The properties of the buffer are conductivity 40μS/cm, relative permittivity 80, density $1000\;kg/m^{3}$, and viscosity 0.001 Pa·s. Three COMSOL physics modules: electric current, creeping flow, and transport of dilute species, were used to conduct numerical analysis. A direct current (DC) source was used to run experiments. Therefore, a stationary study is required to run the modeling. The boundary value conditions for the electric current and the fluid flow motion are reported elsewhere [24,33]. To observe the particle distribution under DEP force, transport of dilute species module is used. For simplicity, the charge of the analyte is considered zero. The analyte solution is governed by the stationary convection–diffusion equation:

(8) $$\nabla \cdot \left(-D\nabla c+vc\right)=R\;,$$

(9) $$v_{x}=u_{x}+\mu_{DEP}\frac{{dE}^{2}}{dx}$$

and

(10) $$v_{y}=u_{y}+\mu_{DEP}\frac{{dE}^{2}}{dy},$$

where D is the dispersive coefficient, c is the concentration of the analyte, v is the velocity of the analyte solution, and R is the source or sink for c. The velocity components $u_{x}$ and $u_{y}$ are from fluid velocity solved from a creeping flow study, *μ_DEP_* is the DEP mobility of the analyte. The inlet is specified by the inflow boundary condition with a desired concentration of the analyte, and the outlet is specified by the outflow boundary condition as follows:

(11) n·D∇c=0.

The side walls are under no flux condition, and the governing equation is:

(12) $$n\cdot \left(-D\nabla c+vc\right)=0.$$

Then, extra fine quality mesh is selected for simulation. The electric current, fluid flow and concentration—all those simulations are under stationary studies. Later, the concentration data of analytes are recorded along a perpendicular line after the 25th gate.

A few deliberate assumptions were made to construct the model to match the experimental condition. The value of the diffusion coefficient was chosen to be three orders of magnitude greater than what would be expected for particles of similar sizes. This was to ensure the appropriate dispersion in the microchannel, i.e., to maintain a similar degree of freedom. The COMSOL module transport of diluted species was used, assuming a homogenous population and equal distribution in a microchannel, which effectively lowered the degree of freedom. A higher concentration was observed in the centerline. With a smaller diffusion coefficient, the species of interest would still be concentrated along the middle line of the channel as the result of the non-uniform electric field but not forming an arc.

### 2.3. Dynamic Range

Electrokinetic and dielectrophoresis mobility data was collected from the literature in DC-iDEP devices (Supplementary Materials). Electrokinetic mobilities were reported via experiment or simulation or approximated through literature-reported values.

Electrokinetic mobility ratio (EKMr) is defined as the ratio of mobilities $\frac{\mu_{EK}}{\mu_{DEP}}$. The lowest potential at which capture occurs (onset potential) is related to the ratio of mobilities, where

(13) $$\frac{\mu_{EK}}{\mu_{DEP}}=\left|\frac{\nabla {\left|E\right|}^{2}}{\left|E\right|}\right|.$$

Finite-element numerical modeling software (COMSOL is used here) can calculate the EKMr value at the onset potential $\frac{\nabla E^{2}}{E}$ given the microchannel geometry and capture location.

## 3. Results

### 3.1. Variance of Onset Voltage from Voltage Sweep

The total variance in g-iDEP measurements (${\sigma^{2}}_{total}$) is the sum of three components: experimental measurement uncertainty (${\sigma^{2}}_{process}$), channel-imposed geometric dispersion (${\sigma^{2}}_{channel-imposed\;limit}$) and natural biological variance (${\sigma^{2}}_{biovariance}$) (Figure 1B). To quantify each component and determine whether g-iDEP can resolve clinically or taxonomically distinct bacterial populations, simulations using reasonable experimental parameters were used to establish these variance components. First the range and standard deviation were established for the voltage sweep method using Monte Carlo calculation, then the channel-imposed limit was determined through finite element analysis and finally these were both compared to the existing literature of bacteria separation and identification.

**Figure 3:**
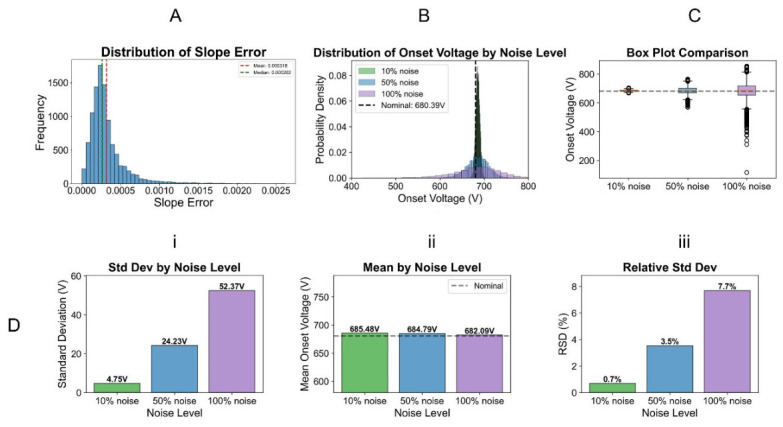
Estimating variance using Monte Carlo simulation introduced by fundamental experimental elements (Equation (7)) that impact EKMr determination by voltage sweep protocol. (**A**) Distribution of slope (‘*a*’ in *y* = *ax* + *b*) variance (*s*, Equation (7)) over 10,000 iterations, with mean (red) and median (green) lines (value ranges detailed in Supplementary Information S1). (**B**) Overlaid probability density distributions of onset voltage for three noise levels artificially added to y (100% = experimentally observed values): 10% (green), 50% (blue), and 100% (purple). Nominal onset voltage, as represented by a single dotted line, is the deterministic value calculated directly from the fitted regression line without considering any uncertainties from the slope, the intercept, in the *x* and *y* measurements. (**C**) Box plot comparison showing medians, quartiles, and outliers for another visualization of the onset voltage distribution. Dotted black line represents the nominal onset voltage. (**D**) Quantitative comparison of onset voltage values, (i) standard deviation (in V), (ii) mean onset voltage, and (ii) relative standard deviation (RSD, in %) across noise levels. All simulations are anchored by experimental data from *S. epidermidis* ATCC 35984 V2L. Raw data and source code can be found in Supplementary Materials.

Bacterial discrimination requires determining whether onset voltage differences between strains/isolates/species exceed the confidence range (Δ*x* > 4σ). Onset voltage is calculated from linear regression (*y* − *b*)/*a*, with slope (*a*) depending on five parameters (Equation (4)): applied voltage, electrokinetic mobility, resistance, charge, and bacterial density. In order to understand the contribution from each parameter to the slope variance, we characterized their variations from the experiment (Table S2): applied voltage showed minimal variation (under 1% RSD), while mobility, resistance, and charge varied by up to 30% RSD. Monte Carlo simulation over 10,000 iterations propagated these uncertainties through Equation (4), revealing low slope variance (represented by σ being under 5 × 10^−4^, or 2% RSD) (Figure 3). The slope variance generated by this method represents variance in electrokinetic mobility rather than dielectrophoretic mobility (Equation (7)) and is smaller in magnitude than the variation in individual contributing parameters.

Considering the low slope variance as a contributing factor, we next examined the standard deviation in the number of bacteria captured (*N*). To assess the impact of experimental error (bacteria counting) on onset voltage, we added Gaussian noise (10%, 50%, 100%) to experimental *y*-values and refit regression lines. Mean onset voltage (680.39 V) remained stable, but standard deviation increased systematically: 4.75 V (0.7% RSD) at 10% noise, 24.23 V (3.6% RSD) at 50%, and 52.37 V (7.7% RSD) at 100% (Figure 3). The 50% scenario represents realistic experimental precision and establishes that bacterial populations with onset voltages differing by 4σ (>96 V) can be reliably distinguished by the voltage sweep method.

### 3.2. Numerical Modeling Variance from Spatial Separation

The channel-imposed limit was investigated with finite element numerical approximations for two device designs (Figure 2) to estimate the variance of the spatial separation. These two specific designs have been used to gather data on cells (detailed below), exosomes [34,35], viruses [36,37], nanoparticles [38] and proteins [39,40].

**Figure 4:**
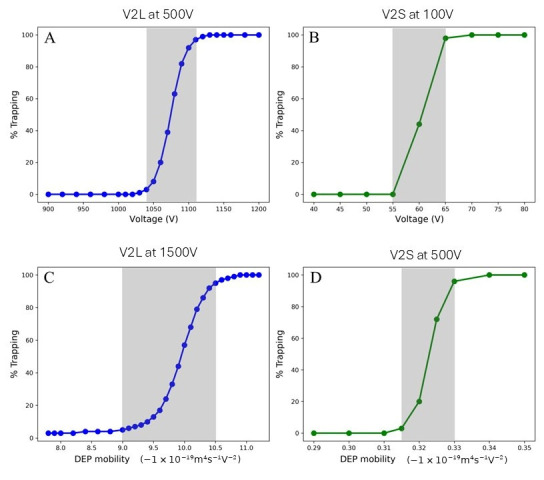
Modeled dielectrophoretic capture behavior of monodisperse particles as a method to estimate the instrument-imposed variance. The percentage of mathematically monodisperse homogeneous population of particles captured at a gate was monitored while varying the applied potential (**A**,**B**) and the mobility of the particles (**C**,**D**) in two device designs: V2L (minimum gap width of 25 μm) and V2S (minimum gap width of 3 μm) (see Figure 2). At intermediate potentials or mobilities (gray zones, **A**–**D**) some particles with identical properties pass through the gate, while others of the exact same type are captured. The inability of the device to induce the same behavior for all identical particles is a metric of the instrument-induced variance and is quantified by noting the range (5–95%) over which partial capture occurs. The particles used in V2L and V2S have different diffusion coefficient values, which are $2.2\times 10^{-10}\;m^{2}s^{-1}$ and $4.4\times 10^{-11}\;m^{2}s^{-1}$, respectively. The ranges are 70 V for V2L (**A**) and 10 V for V2S (**B**) and, as measured by mobility range, are 1.5 × 10^−19^ m^4^s^−1^V^−2^ (**C**) for V2L and 2.0 × 10^−21^ m^4^s^−1^V^−2^ for V2S (**D**).

A model was created using a completely homogeneous mathematically monodisperse population of particles. That model quantified the percentage of particles that passed through a gate area while varying the applied voltage or varying the dielectrophoretic mobility, $\mu_{DEP}$ (Figure 4 and Table 1 and Table 2). The applied voltage and the gate within each device design were chosen to create similar electric field strengths at the gap area in both devices. The diffusion coefficient was also varied to allow the COMSOL MultiPhysics 6.1 to perform the calculations, but these coefficient changes are inconsequential to the motion of the micron-sized particles studied here.

At each gate, some cells or particles will capture near the tips of the inverted triangle at a voltage that is lower than the onset voltage [20,24]. As the voltage is increased, the capture extends towards the center of the channel until a capture occurs at the centerline. Once this threshold is crossed, no more entities of that biophysical property may pass that gate. This model is based on an approach introduced by Keebaugh et al. [41]. There is a small range of applied voltages wherein some particles will pass, and others will be captured, even with completely identical particles.

For the larger-gapped device, the population of identical particles partially passed through the gate area over a range (δ*V*) of several tens of volts (example data: Figure 4A; δV of 40 to 70 V, Table 1, right column) which varied as a function of the *μ_DEP_* of the particle (column 2). For the smaller-gapped device, the same approach generated a smaller range of δV (15–30 V) (Figure 4B). At a set voltage, the range of particles as defined by their dielectrophoretic mobility (*μ_DEP_*) values which partially captured the gate area ranged from 0.3 to 1.5 (−1 × 10^−19^ m^4^s^−1^V^−2^) for the larger-gapped design (V2L) (Figure 4C, Table 2) and about an order of magnitude smaller for the smaller gates (V2S): 0.2 to 6 (−1 × 10^−20^ m^4^s^−1^V^−2^) (Figure 4D, Table 2).

Similarly, a specific value of *μ_DEP_* was explored in the range of $10^{-19}$ to $10^{-18}$ m^4^s^−1^V^−2^ (Table 1, column 2). At the same *μ_DEP_* of ${-4\times 10^{-19}\;m}^{4}s^{-1}V^{-2}$, the transition zone of V2L (70 V) is more than 4 times larger than that of V2S (15 V). The chosen *μ_DEP_* values are about a magnitude smaller for V2S, and the transition zone is approximately two times smaller than V2L.

**Figure 5:**
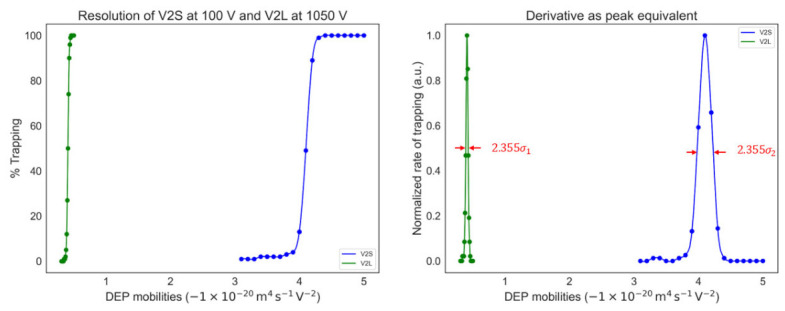
Direct comparison of percent trapped for V2S and V2L (**left**) and rate of change for trapping (**right**) versus $\mu_{DEP}$. The voltages selected for V2L (1050 V) and V2S (100 V) create equal electric field gradients at the gaps. The rate of change or derivative of percent trapping (**right**) provides a quantifiable and direct metric of channel-induced variance of g-iDEP devices as a function of DEP mobility. The initial data form is interpreted as an Error Function and the derivative is modeled as a Gaussian distribution where the full-width at half maximum allows the determination of the variance ($\sigma^{2}$) for each σ.

The first derivative was calculated and plotted from the percent captured data (Figure 5 (right)). The data structure is assumed to be in the form of an Error Function such that the resulting derivative approximates a Gaussian distribution with associated standard deviation (σ) and variance ($\sigma^{2}$). As a result, for all these conditions, a specific quantified value of the standard deviation (therefore range and variance) in applied voltage (units of volts) and *μ_DEP_* (units of m^4^s^−1^V^−2^) is available.

### 3.3. Dynamic Range Visualization

**Figure 6:**
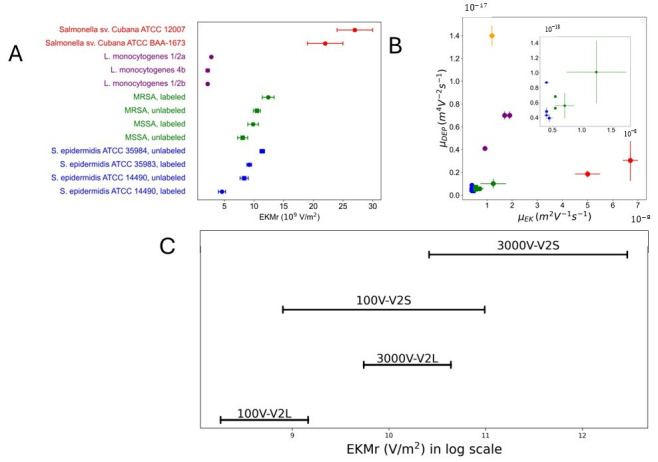
Visualization of the dynamic range for existing g-iDEP device designs and multiple species of bacteria. Current separation based on previously reported values for multiple bacterial genera (*Salmonella*, *Listeria* *monocytogenes*, and *Staphylococcus*), including their subsequent species- and strain-level samples, is shown using (**A**) the electrokinetic mobility ratio (EKMr) and (**B**) combined DEP and electrokinetic (EK) mobilities. In both representations, bacterial strains cluster according to phylogenetic relatedness, with improved separation in (**B**) due to the two-dimensional mobility space. Isolates are color-coded by species (red: *Salmonella*, purple: *L. monocytogenes*, green: *S. aureus*, blue: *S. epidermidis*) for visualization. (**C**) Estimated dynamic range of EKMr achievable with the current device design, corresponding to applied voltages from 100 V to 3000 V and spanning approximately four orders of magnitude. Experimental and analytical methods are described in Supplementary Materials.

Experimental data across several genus, species and strains have been collected and published using the same device design (and one very similar one, from earlier works) [21,22,23,27]. The absolute value and the range of values for each data set have been tabulated (Tables S4 and S5) and plotted (Figure 6). The difference in biophysical properties of the closest strains have been proven sufficient for differentiation for *Escherichia coli* (O6:K1:H1, O55:H7 and O157:H7) [42], *Staphylococcus epidermidis* (labeled, differentiated: gentamicin resistant ATCC 35983 and sensitive ATCC 14990) [23,43,44], *Listeria monocytogenes* (serovars 1/2a, 1/2b, and 4b) [21], *Staphylococcus aureus* (methicillin-resistant ATCC 43300 (MRSA) and methicillin-sensitive ATCC 29213 (MSSA)) [22], *Salmonella* (sv. *Cubana*, sv. *Poona*) [27] and carbapenemase ((KPC)-positive TG60131 and TG40855; KPC-negative TG9347) [45]. All of these studies focused on the most closely related strains, isolates, or serovars as measured by traditional typing strategies (culture/metabolism, sequencing, phenotyping, etc.). When represented using the electrokinetic mobility ratio (Figure 6A), bacterial taxa form distinct clusters that broadly reflect phylogenetic relatedness at the genus. However, overlap between closely related strains limits discrimination in the one-dimensional EKMr representation. In contrast, mapping the same data in a two-dimensional mobility space defined by DEP and electrokinetic mobilities (Figure 6B) improves separation, yielding more resolved strain-level clustering. The achievable operating range of the current device design is shown in Figure 6C. By varying the applied voltage from 100 V to 3000 V, the accessible EKMr spans approximately four orders of magnitude, proving a broad dynamic range capable of encompassing the reported mobility values across multiple bacterial genera and strains.

## 4. Discussion

### 4.1. Comparing the Estimated Range and the Microchannel-Imposed Limit

Considering the same design of V2L and representative parameters of bacterial cells, the 4σ voltage range for reliable differentiation is 96 V (voltage sweep) and 40–70 V (spatial differentiation), corresponding to EKMr values of 9.18–10.6 × 10^9^ and 0.582–1.02 × 10^9^ V/m^2^, respectively. With a much narrower gap in V2S, the range is significantly reduced to 15–30 V (EKMr: 0.542 to 1.08 × 10^9^ V/m^2^). As seen in Figure 4 and Table 1, the resolution improves with higher voltage (i.e., higher field gradient), but even at equivalent field gradient strength, the V2S design with a narrower constriction gap still has a smaller range compared to that of V2L (Figure 5). Spatial separation mode [23,34] is therefore expected to offer approximately three-fold greater resolving power than the voltage sweep approach.

These results highlight a fundamental distinction between the two measurement approaches: spatial differentiation is dependent on the bioparticle dispersion across the gap area, and hence, the microchannel geometry, while the differentiation via voltage sweep is primarily determined by the electrokinetic mobility effects and cell count variance. For the spatial method, as the gap between insulators widens, the difference in EKMr increases between the centerline and the tip region at a given voltage. This spatial variation in EKMr across the gate area, from tip to tip, contributes to the channel-imposed limit, as analytes on different path lines experience different effective forces [24].

The result of the Monte Carlo simulations which investigates the source of the variance rather than the total variance does align with all past bacterial differentiations including *E. coli*, *S. aureus*, *Salmonella*, *Klebsiella pneumoniae* and *Listeria* [21,22,23] in that the onset voltage changed by about 100 V using the voltage sweep method for all paired strain (variant, isolate, serovar, etc.) experiments. This study suggests the majority of the variance in those determinations is the cell count and electrokinetic mobility effects rather than any intrinsic limitation of the strategy.

### 4.2. Connecting the Sources of Variance

**Figure 7:**
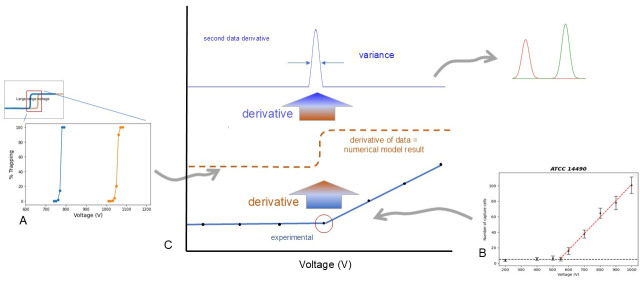
Visualizing the variance from models and experimental results using the potential sweep method of EKMr determination. (**A**) Representation of large voltage range numerical simulations plotted as percentage trapped versus voltage and finite element simulation data focused on the transition zone (see Figure 5). (**B**) Representative data used as a visual aid showing the basis for obtaining derivative from experimental results. (**C**) Graphic showing that the first derivative of the experimental data is equivalent to numerical models plotted as percentage trapped. Taking the derivative of the numerical model (Figure 5) and the second derivative of the experimental data gives a peak with a characteristic variance which can be compared favorably to classic separations such as isotachophoresis [46].

Plotting intensity or number of particles versus applied potential has a finite transition about the onset potential from zero slope to a new constant value (Figure 7B representing published data, red circle in Figure 7C). The first derivative of a fine-scale experimental data set, say captured at one-to-ten-volt increments, would have a sigmoidal shape similar to the transitions generated in the numerical modeling presented here. From either of these two sources (finite element model or slope of the data), a derivative may be created to generate a peak-like structure which can be interpreted as chromatography peaks (or population distributions) with all of the advantages and properties of known distribution statistics and mathematics.

### 4.3. Existing Data Sets and Their Variance

The work presented above focuses on the voltage increment at a single gate (or an insular constriction), where the electric field gradient is at its maximum. Previously, spatial differentiation was also reported, such as the dual-strain, simultaneous capture of gentamicin-resistant (red) and gentamicin-susceptible (green) *S. epidermidis* within separate regions of a single microchannel [23] or separation of the sub-population of neural stem and progenitor cells that are either neuron- or astrocyte-biased [28]. In both separation schemes, resolution is governed by the strength of the electric field gradient along the centerline, which is inherently constrained by the microchannel geometry. Spatial separation enables simultaneous discrimination of analytes but requires sufficient differences in EKMr across successive gates at a fixed voltage. The dynamic range is further limited by the total number of gates. Incremental voltage application improves quantification; however, as shown in the modeling results, the trajectory of particles will differ away from the centerline, creating variance.

### 4.4. Limitations and Improvements

This work assumes linear electrokinetic (electrophoretic and electroosmosis) behavior. At sufficiently high electric field strengths, nonlinear electrokinetic effects (e.g., nonlinear electrophoresis and electroosmosis) may become significant [47,48] and are not captured in the present model. Future studies can extend this framework to incorporate nonlinear electrokinetic effects, particularly under high-field conditions where such mechanisms may influence particle transport and separation performance in gradient insulator-based systems.

The physical device used in these experiments is understood to be a relatively inefficient system, enough to achieve separation between the species and isolates under a genus but incapable of realizing the ideal high-resolution, low-variance separations of DEP [24]. The calculated imposed limit of the microchannel can be compared favorably to the theoretical limits of this strategy [9]. This study generated *μ_DEP_* on the order of 10^−20^ m^4^V^−2^s^−1^ with experimental estimations largely agreeing with the calculated values. Jones and Hayes [9] suggest that under reasonable experimental parameters, a microchannel with improved gate area designs could easily generate a minimum *μ_DEP_* of 10^−23^ m^4^V^−2^s^−1^. Under ideal experimental parameters this value can be as small as 10^−26^ m^4^V^−2^s^−1^.

Alternative image processing strategies can reduce the variance in existing device designs. By only quantitating the centerline of the capture zone, focusing on the last section of the center of the arc for capture behavior, the variance can be reduced. When imaging the entire channel, identifying the last gate of penetration can also reduce variance (similar strategy to the centerline quantification). These two strategies are estimated to reduce variance by one-half to one order of magnitude. Future research should be aimed at optimization of microfluidic device designs and experimental protocols. By fully exploiting the higher resolution promised by dielectrophoresis, we can begin to quantify the innate biophysical properties and temporal cell behavior or responses, for example, during different cell cycles. Single-cell interrogation by dielectrophoresis will also be possible, allowing further investigation into cell heterogeneity and cell sub-populations.

## 5. Conclusions

This work establishes a quantitative framework for understanding resolution limits for experimental gradient insulator-based dielectrophoresis through systematic variance decomposition. Two methods, voltage sweep and spatial, have been used to determine EKMr values in two device designs (V2S and V2L). The range (4σ, Δx≥ 4σ) for a given population of particles has been examined for these two methods in these two designs, thus providing common metrics in the separations science community of variance ($\sigma^{2}$), standard deviation (σ) and FWHM (2.35σ). These are described in terms of onset voltage, EKMr values and $\mu_{DEP}$, to quantitatively compare the overall strategy with other separations methods. In addition, the full range of available EKMr values accessible with existing designs with reasonable electric fields has been determined and therefore the dynamic range established. These experimental values can be quantitatively compared to theoretical limits.

Most EKMr values have been estimated using the voltage sweep method. Monte Carlo simulations showed that variances from cell count and electrokinetic effects were dominant. The study centered on common experimental conditions resulted in a range of 4σ= 96 V (630 V–730 V in V2L, EKMr: 9.18 to 10.6 × 10^9^ V/m^2^), aligning well with close-pair differentiations from the literature (Δx~100 V). Separately, finite element analysis revealed that channel geometry constrained resolution under any conditions, even using mathematically monodisperse samples and quantifying EKMr with the spatial method. The range (4σ) over which capture partially occurs is 40–70 V (V2L) (EKMr: 0.582 to 1.02 × 10^9^ V/m^2^; *δμ_DEP_* from −0.3 to 1.5 ×10^−19^m^4^s^−1^V^−2^) and 15–30 V (V2S) (EKMr: 0.542 to 1.08 × 10^9^ V/m^2^; *δμ_DEP_* from −02 to 0.6 ×10^−19^m^4^s^−1^V^−2^). These results indicate that an experiment performed in the spatial mode will have about 3× (1.4 to 6× expressed as voltage range) the resolving power compared to the voltage sweep mode.

This computational study generated *δ_DEP_* on the order of 10^−20^ m^4^V^−2^s^−1^ with experimental results largely agreeing with the calculated values. Jones and Hayes [9] suggested that under reasonable experimental parameters, a microchannel with improved gate area designs as presented in Crowther and Hayes [24] could easily generate a minimum *μ_DEP_* of 10^−23^ m^4^V^−2^s^−1^. Under ideal experimental parameters this value can be as small as 10^−26^ m^4^V^−2^s^−1^. Other constraining factors will come into play when these high-resolution strategies are developed, including the accuracy and reproducibility of fabricating the devices and the stability of the power supplies. While this strategy already demonstrated fairly extraordinary capabilities to differentiate important unlabeled bioparticles including close-paired bacteria strains, these results suggest there is still a large opportunity (three to six orders of magnitude) for further improvements.

## Figures and Tables

**Table 1:** Comparisons for different devices estimating the smallest range of applied voltage for moving from particle passage to complete capture. The choice of *μ_DEP_* was based on prior research, where most particles were found to fall within the $10^{-19}$ to $10^{-18}$ m^4^s^−1^V^−2^ range [20,24,39]. Increased diffusion coefficients were used to ensure numerical stability in COMSOL, with negligible impact on results for the micron-sized particles used in this study.

Device Type	$\mu_{DEP}$$\;({-10}^{-19}$ m^4^s^−1^V^−2^)	Diffusion Coefficient Used in Modeling, *D* (m^2^/s)	δV (V)
V2L	4	$2.2\times 10^{-10}$	70
	8		50
	12		40
V2S	0.8	$4.4\times 10^{-11}$	30
	1		25
	4		15

**Table 2:** Summary of estimated minimum resolvable differences in dielectrophoretic mobility (δ) for V2L and V2S. Each voltage was selected within a practical range based on prior studies [36,37]. Increased diffusion coefficients were used to ensure numerical stability in COMSOL, with negligible impact on results for the micron-sized particles used in this study.

Device Type	Voltage (V)	Diffusion Coefficient Used in Modeling, *D* (m^2^/s)	$\delta \mu_{DEP}$ (−1 × 10^−19^ m^4^s^−1^V^−2^)
V2L	500	$2.2\times 10^{-10}$	1.5
	1000		0.4
	1500		0.3
V2S	100	$4.4\times 10^{-11}$	0.6
	300		0.07
	500		0.02

## Data Availability

The data that support the findings of this study are available from the corresponding author upon reasonable request.

## Supplementary Materials

The following supporting information can be downloaded at: https://www.mdpi.com/article/10.3390/mi17050600/s1, Supplementary Information S1: Estimation of variance in slope and onset voltage. Supplementary Information S2: *Separation bacteria* of different species and isolates by electrokinetic mobility (EKMr), electrokinetic mobility μ_EK_ and dielectrophoretic mobility μ_DEP_. Figure S1: Voltage variability observed with common surface treatments, including 0.4% BSA, 1% PVA, and 1% PEG. Voltage was sampled at 16 readings per second, and a 10-s average was computed for each microdevice as voltage was applied. For both the V2L (left) and V2S designs (right), the mean values from six microdevices at each voltage setting were recorded and plotted. Figure S2: Charge variability observed with common surface treatments, including 0.4% BSA, 1% PVA, and 1% PEG. Charged a 10-s average was computed for each microdevice during the applied voltage period. For both the V2L (left) and V2S (right) designs, the mean values from six microdevices at each voltage setting were recorded and plotted. Figure S3: Determination of electrokinetic mobility as a slope of the linear regression between electric field strength and particle velocity. The electric field strength corresponds to V2L design, at voltage application of 100 to 500 V at the runway, where electric field is linear. Particle tracking was performed for 3 particles at each voltage, and the velocity values were averaged and plotted. Only the data for BSA pre-treatment is available. Figure S4: Monte Carlo simulation results for slope error between electrokinetic mobility (EK), resistance (R), and charge (Q) as correlated parameters and bacteria density (n) as independent parameter (a) Probability density of slope errors across 10,000 simulations for independent (gray) and correlated EK/R/Q under weak (blue), moderate (red), and strong (orange) correlations. Stronger correlations increase variability and shift the distribution slightly (b) Heatmaps of the correlation matrices between EK, R, and Q for weak, moderate, and strong correlation, illustrating the expected positive and negative relationships driven by conductivity dependence. Figure S5: Linear regression of bacteria counts vs. voltage in the 700–1000V range with error bars, showing the fitted line (purple) and 90% confidence intervals for each noise condition as shaded regions. Horizontal green dashed line indicates the threshold (y = 2 bacteria); vertical blue dashed line is the nominal onset voltage. Table S1: Summary of the variability in conductivity across the common surface treatments (0.4% BSA, 1% PVA, and 1% PEG) for both the V2L and V2S designs. Conductivity was determined from the slope of the voltage–current linear regression, and the reported values represent the mean from six microdevices for each treatment condition. As conductivity is inversely proportional to the resistance of the microchannel, the deviation and variance values of conductivities can be used to estimate the deviation and variance values of the resistance. Table S2: Summary of the range of variance used in Monte Carlo simulation. Table S3: Summary of result for variance of onset voltage by adding Gaussian noise at 10, 50 and 100%. Mean, Standard Deviation and 90% Confident Interval were reported in voltage. The weighted linear regression slope is 0.3617 ± 0.0587, intercept is -244.12 ± 43.40. The onset voltage without Gaussian is 701.49 ± 183.24 V. Table S4: EKMr value of *Staphylococcus epidermidis*, *Staphylococcus aureus*, *Listeria moncytogenes* and *Salmonella*. Table S5: Electrokinetic mobility and dielectrophoretic mobility of *Staphylococcus epidermidis*, *Staphylococcus aureus*, *Listeria moncytogenes* and *Salmonella*.

## Abbreviations

The following abbreviations are used in this manuscript:

DEP	Dielectrophoresis
g-iDEP	Gradient insulator-based dielectrophoresis
EK	Electrokinetics
EP	Electrophoresis
DC	Direct current
EKMr	Electrokinetic mobility ratio
