# Supplementary material for: Quantifying and Minimizing the Variance of Gradient Insulator-Based Dielectrophoresis
Source: Micromachines (Basel). 2026 May 14;17(5):600. doi: 10.3390/mi17050600 (PMC13208963; doi:10.3390/mi17050600)
Supplement: Supplementary file 1 [file micromachines-17-00600-s001.zip › micromachines-4282592-supplementary.pdf]

Supplementary Information S1: Estimation of variance in slope and onset voltage

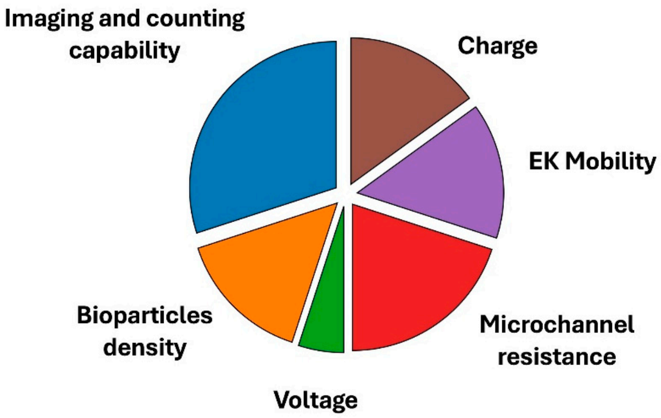

1. Determination of the experimental variance

The devices were prepared by depositing a mixture of polydimethylsiloxane (PDMS) (Sylgard 184, Dow/Corning, Midland, MI) and the curing agent onto the template wafers, then transferred to the oven and baked for one hour at 70°C. Afterward, the PDMS casts were removed from the wafer, trimmed, and stored in a 4°C freezer for up to one month before use. Each DEP device, which consists of the PDMS cast and glass slides bonded by oxygen plasma, was prepared on the day of the experiment. A biopsy needle was used to create openings in the PDMS to form inlet and outlet reservoirs. Before bonding, the glass slides were cleaned using an alkaline glass-cleaning solution (Hellmanex® III, Hellma, Germany), acetone, and isopropyl alcohol, sonicated for 30 s, and rinsed with DI water. The PDMS casts were cleaned with isopropyl alcohol and DI water only to avoid swelling of the polymeric material. The PDMS and glass were treated with oxygen plasma using a plasma cleaner (Zepto, Diener, CA) for 30 seconds at 18 W. The PDMS and glass were then sealed by contact where a seal occurs instantaneously. The microdevices were used on the day they were bonded.

For the dielectrophoretic determination of bioparticles using voltage sweep, *Staphylococcus epidermidis* ATCC 35984 was used. Experiments were conducted in a BSL II laboratory following Institutional Biosafety Committee (IBC) and National Institute of Health (NIH) guidelines. Lyophilized sample was purchased from American Type Culture Collection (ATCC) and revived in a tryptic soy broth (TSB) to generate seed stock. The seed stock was streaked onto agar, and incubated overnight at 37°C, and diluted to OD 600 of 0.3 in the phosphate buffer.

Three common surfactants for PDMS in phosphate buffer 2 mM were tested for stability testing: 4 mg/ml BSA, 1% (w/v) polyvinyl alcohol (PVA), 1% (w/v) Polyethylene glycol. A total of 10 ul of the chosen buffers with surfactants were pipetted into the device and were left to sit for 15 minutes, and pipetted out, followed by several phosphate buffer washes to minimize the free-flowing surfactant.

A 10 µL volume of the 2 mM phosphate buffer was added to the inlet and outlet reservoirs. Platinum electrodes (Alfa Aesar, Ward Hill, MA) connected to a high-voltage sequencer (HVS448 3000D, Labsmith, CA) were inserted into both reservoirs. A DC voltage was applied stepwise from 100 V to 1000 V in 100 V increments, each held for 10 seconds. To mitigate hydrodynamic flow, phosphate buffer was replenished between voltage steps as needed.

The values of voltages and respective currents were monitored through the high-voltage sequencer. The resistance was determined through the inverse slope of the voltage–current linear regression. The charge was calculated as the total current in a duration of 10 seconds, which is typical of the applied voltage .

The onset voltage was determined from the linear regression of x (voltage) and y (number of cells captured, whether via counting individual cells or by intensity)

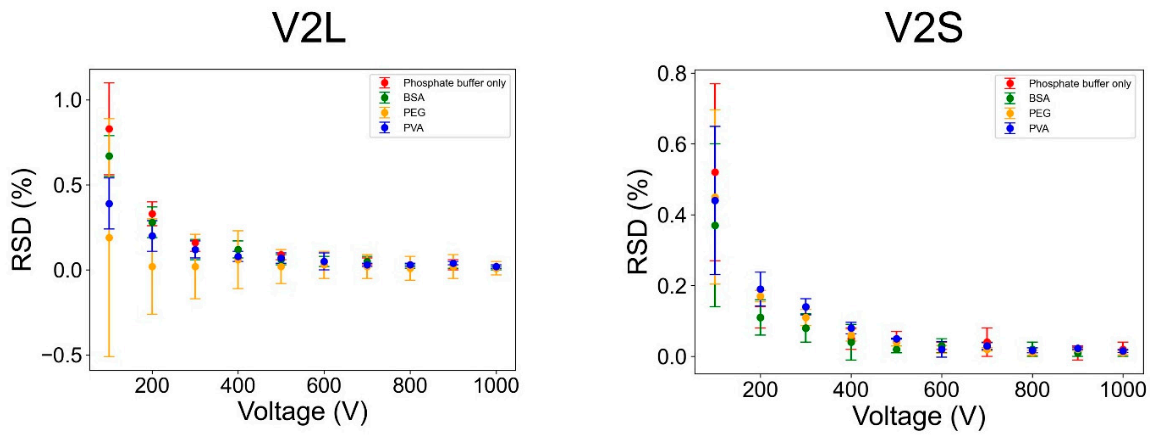

Figure S1: Voltage variability observed with common surface treatments, including 0.4% BSA, 1% PVA, and 1% PEG. Voltage was sampled at 16 readings per second, and a 10-second average was computed for each microdevice as voltage was applied. For both the V2L (left) and V2S designs (right), the mean values from six microdevices at each voltage setting were recorded and plotted.

There is little deviation from voltage application, as seen here in less than 0.01 (or 1%), both in V2S and V2L across multiple device pre-treatments. At a lower voltage, the variance is slightly higher, but given that bacteria cells got captured at 400V and above, these values are under 0.002 (or 0.2%). Hence, the range of standard deviation was chosen to be (0; 0.002) and (0; 4 x 10^{-6})

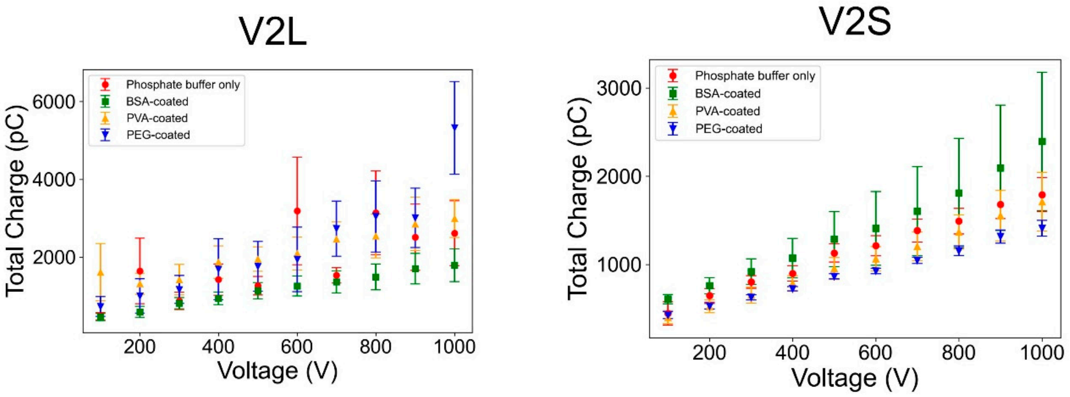

Figure S2: Charge variability observed with common surface treatments, including 0.4% BSA, 1% PVA, and 1% PEG. A 10-second average was computed for each microdevice during the applied voltage period. For both the V2L (left) and V2S (right) designs, the mean values from six microdevices at each voltage setting were recorded and plotted.

As seen here, the standard deviation of charge varies from 0.1 to 0.2 (or 10 - 30%), and the range of variance can be estimated to be in the range of (0.01 to 0.09).

Table S1: Summary of the variability in conductivity across the common surface treatments (0.4% BSA, 1% PVA, and 1% PEG) for both the V2L and V2S designs. Conductivity was determined from the slope of the voltage–current linear regression, and the reported values represent the mean from six microdevices for each treatment condition. As conductivity is inversely proportional to the resistance of the microchannel, the deviation and variance values of conductivities can be used to estimate the deviation and variance values of the resistance

	No treatment	BSA (0.4%)	PVA 1%	PEG (1%)
V2L (uS)	0.0091 ± 0.0029	0.0113 ± 0.0024	0.0137 ± 0.0029	0.0292 ± 0.0036
RSD %	31%	21%	21%	12%
V2S (uS)	0.009 ± 0.001	0.011 ± 0.002	0.0079 ± 0.0007	0.0068 ± 0.0006
RSD %	11%	18%	8.8%	8.8%

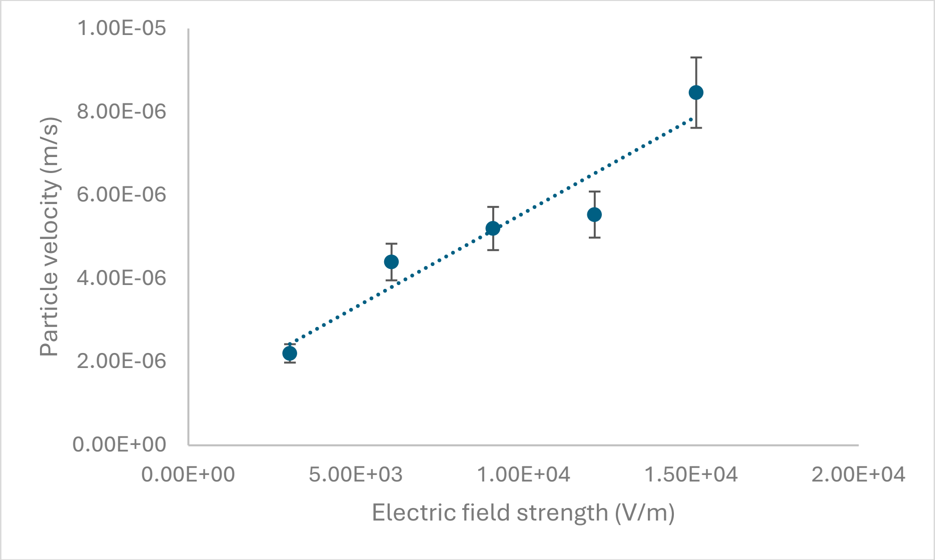

Figure S3: Determination of electrokinetic mobility as a slope of the linear regression between electric field strength and particle velocity. The electric field strength corresponds to V2L design, at voltage application of 100 to 500 V at the runway, where electric field is linear. Particle tracking was performed for three particles at each voltage, and the velocity values were averaged and plotted. Only the data for BSA pre-treatment is available.

The variability of the electrokinetic (EK) response was quantified by estimating the uncertainty in the slope of the regression relating electric field strength to particle velocity. Because particles near the channel walls may experience a different effective zeta potential compared with those traveling near the centerline, particle-tracking measurements were performed on multiple trajectories to capture this spatial heterogeneity. Across three independent experiments (Figure S3), the standard deviation of the measured EK mobility was consistently below 0.05 (≈5%). Therefore, the estimated range of variance is (0; 0.0025).

The particle count within the microchannel was estimated from OD_{600} measurements, which provide an approximate proxy for colony-forming units (CFU) in bacterial suspensions. Although OD_{600} does not perfectly reflect viable counts, the concentration differences relevant to these experiments remain within a single order of magnitude. Based on typical OD_{600} ranges used and the observed relationship between onset voltage and the number of captured particles, the effective particle concentrations can be reasonably constrained to approximately 0 – 0.25 (up to to 25% relative deviation), or in the variance range of (0 – 0.055). This range consists of empirical behavior during capture experiments and provides a practical estimate for modeling the resulting variability in onset behavior.

2. Simulation of the slope variance using Monte Carlo

Table S2: Summary of the range of variance used in Monte Carlo simulation

	Standard deviation		Variance	
	Low	High	Low	High
Voltage	0	0.002	0	4.00×10^{-6}
n	0	0.25	0	0.055
R	0.1	0.3	0.01	0.09
EK	0	0.05	0	0.0025
Q	0.1	0.3	0.01	0.09

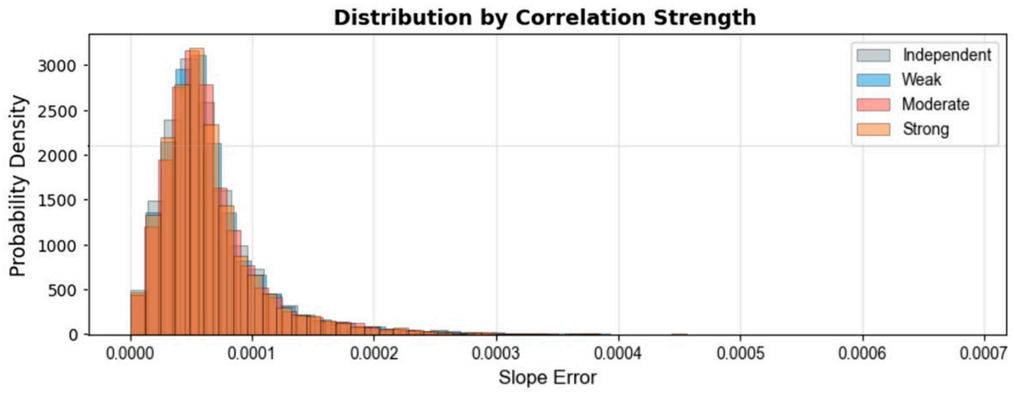

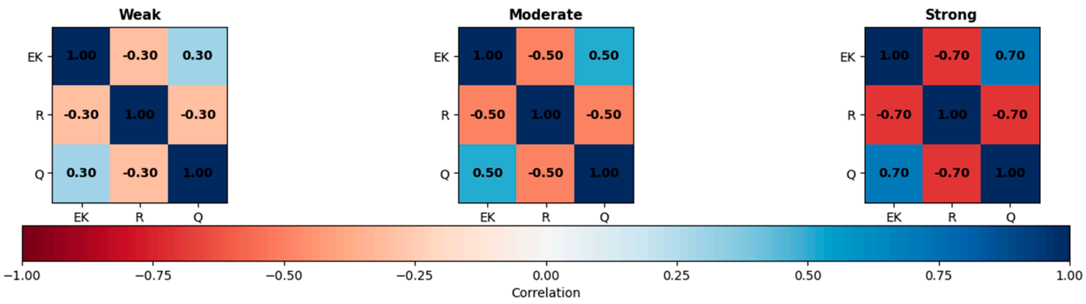

Figure S4: Monte Carlo simulation results for slope error between electrokinetic mobility (EK), resistance (R), and charge (Q) as correlated parameters and bacteria density (n) and as independent parameter (a). Probability density of slope errors across 10,000 simulations for independent (grey) and correlated EK/R/Q under weak (blue), moderate (red), and strong (orange) correlations. Stronger correlations increase variability and shift the distribution slightly. (b) Heatmaps of the correlation matrices between EK, R, and Q for weak, moderate, and strong correlation, illustrating the expected positive and negative relationships driven by conductivity dependence.

Three correlation levels were tested for R, EQ and K: weak (0.3), medium (0.5) and strong (0.7), as shown in Figure S4. The difference between the uncertainty of the slope is negligible between correlation status. The slope uncertainty averages 0.00025, with 95% falling under 0.001.

Monte Carlo Python source code

```python
import numpy as np
import matplotlib.pyplot as plt
from scipy.stats import multivariate_normal
from dataclasses import dataclass


# CONFIGURATION

@dataclass
class SimulationConfig:
    # Monte Carlo
    n_samples: int = 20
    num_sims: int = 10_000

    # Bacteria count
    N_min: float = 10
    N_max: float = 1000

    # Voltage variance
    var_x_min: float = 0.0
    var_x_max: float = 4e-6

    # n (independent)
    var_n_min: float = 0.01
    var_n_max: float = 0.055

    # EK, R, Q (correlated)
    var_ek_min: float = 0.01
    var_ek_max: float = 0.0025

    var_r_min: float = 0.01
    var_r_max: float = 0.09

    var_q_min: float = 0.01
    var_q_max: float = 0.09

    # Correlation strength: 'weak', 'moderate', 'strong'
    correlation_strength: str = "moderate"


# CORRELATION MODEL


def create_correlation_matrix_EK_R_Q(strength: str):
    base = {"weak": 0.3, "moderate": 0.5, "strong": 0.7}.get(strength, 0.5)

    return np.array([
        [1.0,    -base,   base],
        [-base,   1.0,   -base],
        [base,   -base,   1.0]
    ])


def sample_correlated_EK_R_Q(means, stds, corr_matrix):
```

```python
    cov = np.diag(stds) @ corr_matrix @ np.diag(stds)
    return multivariate_normal.rvs(mean=means, cov=cov)

# MONTE CARLO SIMULATION

def monte_carlo_slope_error(cfg: SimulationConfig):
    corr_matrix = create_correlation_matrix_EK_R_Q(cfg.correlation_strength)

    slope_corr = []
    slope_indep = []

    # Precompute EK/R/Q distribution parameters
    means = np.array([
        (np.sqrt(cfg.var_ek_min) + np.sqrt(cfg.var_ek_max)) / 2,
        (np.sqrt(cfg.var_r_min)  + np.sqrt(cfg.var_r_max))  / 2,
        (np.sqrt(cfg.var_q_min)  + np.sqrt(cfg.var_q_max))  / 2
    ])

    stds = np.array([
        (np.sqrt(cfg.var_ek_max) - np.sqrt(cfg.var_ek_min)) / 4,
        (np.sqrt(cfg.var_r_max)  - np.sqrt(cfg.var_r_min))  / 4,
        (np.sqrt(cfg.var_q_max)  - np.sqrt(cfg.var_q_min))  / 4
    ])

    for _ in range(cfg.num_sims):
        N = np.random.uniform(cfg.N_min, cfg.N_max)
        var_x = np.random.uniform(cfg.var_x_min, cfg.var_x_max)

        mean_n = (np.sqrt(cfg.var_n_min) + np.sqrt(cfg.var_n_max)) / 2
        std_n  = (np.sqrt(cfg.var_n_max) - np.sqrt(cfg.var_n_min)) / 4
        var_n  = abs(np.random.normal(mean_n, std_n))**2

        # Correlated EK/R/Q
        ek, r, q = abs(sample_correlated_EK_R_Q(means, stds, corr_matrix))**2
        var_y_corr = N * np.sqrt(var_n**2 + ek**2 + r**2 + q**2)

        slope_corr.append(
            np.sqrt(1 / (cfg.n_samples - 2)) * np.sqrt(var_y / var_x_corr)
        )

        # Independent EK/R/Q
        ek_i = abs(np.random.normal(means[0], stds[0]))**2
        r_i  = abs(np.random.normal(means[1], stds[1]))**2
        q_i  = abs(np.random.normal(means[2], stds[2]))**2
        var_y_indep = N * np.sqrt(var_n**2 + ek_i**2 + r_i**2 + q_i**2)

        slope_indep.append(
            np.sqrt(1 / (cfg.n_samples - 2)) * np.sqrt(var_x / var_y_indep)
        )

    return {
        "correlated": np.array(slope_corr),
        "independent": np.array(slope_indep),
        "corr_matrix": corr_matrix,
        "strength": cfg.correlation_strength
    }
```

```python
# CORRELATION STRENGTH COMPARISON

def compare_correlation_strengths(cfg: SimulationConfig):
    results = {}

    for strength in ["weak", "moderate", "strong"]:
        cfg_local = SimulationConfig(**vars(cfg))
        cfg_local.correlation_strength = strength
        results[strength] = monte_carlo_slope_error(cfg_local)

    results["independent"] = {
        "independent": results["weak"]["independent"]
    }

    return results


def print_summary(results):
    print("\nSLOPE ERROR SUMMARY (n independent, EK/R/Q correlated)")
    print("-" * 80)
    print(f"{'Case':<12} {'Mean':<12} {'Std':<12} {'5-95%':<12}")
    print("-" * 80)

    indep_std = np.std(results["independent"]["independent"])

    for key in ["independent", "weak", "moderate", "strong"]:
        data = (
            results[key]["independent"]
            if key == "independent"
            else results[key]["correlated"]
        )
        mean = np.mean(data)
        std  = np.std(data)
        ci   = np.percentile(data, 95) - np.percentile(data, 5)

        print(f"{key:<12} {mean:<12.6f} {std:<12.6f} {ci:<12.6f}")

    print("-" * 80)
    print("Impact of ignoring EK/R/Q correlations:")
    for key in ["weak", "moderate", "strong"]:
        diff = np.std(results[key]["correlated"]) - indep_std
        print(f"{key:<10}: ΔStd = {diff:+.6f} ({diff/indep_std:+.1%})")
    print("-" * 80)


# MAIN

if __name__ == "__main__":

    cfg = SimulationConfig(
        var_x_max=1e-4,
        var_ek_min=0.0,
        var_ek_max=0.0025
```

```
)

results = compare_correlation_strengths(cfg)
print_summary(results)

np.savez(
    "slope_error_partial_correlation.npz",
    independent=results["independent"]["independent"],
    weak=results["weak"]["correlated"],
    moderate=results["moderate"]["correlated"],
    strong=results["strong"]["correlated"],
)

print("\nResults saved to slope_error_partial_correlation.npz")
```

3. Simulation of the variance of onset voltage

With the estimated slope, onset voltage was then determined as (y-b)/a. Gaussian noise at three different levels (0%, 50% and 100%) was added to the recorded bacteria number (y). Using sample data of a spherical bacteria isolate (*Staphylococcus epidermidis* ATCC 35984), a distribution of onset voltage was determined. Gaussian noise at three different levels (0%, 50% and 100%) was added to the recorded bacteria number (y). The original data can be found under the source code. The summary for variance in onset voltage depending on Gaussian noise is presented in Table S3, with the source code provided after.

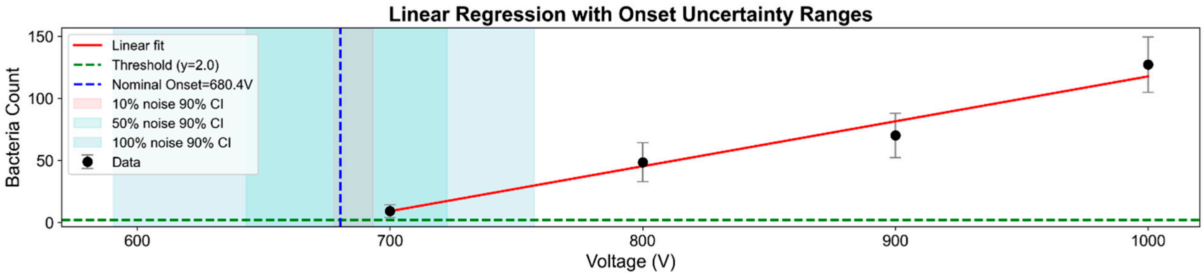

Figure S5: Linear regression of bacteria counts vs. voltage in the 700–1000V range with error bars, showing the fitted line (purple) and 90% confidence intervals for each noise condition as shaded regions. Horizontal green dashed line indicates the threshold (y=2 bacteria); vertical blue dashed line is the nominal onset voltage.

Table S3: Summary of result for variance of onset voltage by adding Gaussian noise at 10, 50 and 100%. Mean, standard deviation and 90% confidence interval are reported in voltage. The weighted linear regression slope is 0.3617 ± 0.0587, intercept is -244.12 ± 43.40. The onset voltage without Gaussian is 701.49 ± 183.24 V.

Noise level (%)	Mean (V)	Std Dev (V)	RSD (%)	90% CI Width (V)
10	685.52	4.78	0.7	15.71
50	684.51	24.20	3.5	78.7
100	681.57	52.17	7.7	167.97

Source code for variance of onset voltage

```
import numpy as np
import matplotlib.pyplot as plt
from scipy import stats
from scipy.optimize import curve_fit
from dataclasses import dataclass


# CONFIGURATION


@dataclass
class OnsetConfig:
    # Original data
    x_values: np.ndarray = np.array([200, 400, 500, 600, 700, 800, 900,
1000])
    y_values: np.ndarray = np.array([2.33, 2.60, 3.17, 2.17, 9.17, 48.58,
70.08, 127.16])
    y_errors: np.ndarray = np.array([1.41, 1.57, 1.66, 2.38, 5.16, 15.62,
17.86, 22.28])

    # Linear region selection
    linear_min: float = 700

    # Onset threshold
    y_threshold: float = 2.0

    # Monte Carlo options
    num_sims: int = 10_000
    noise_levels: list = (0.1, 0.5, 1.0)  # as fraction of error bars (10%,
50%, 100%)


def select_linear_region(cfg: OnsetConfig):
    mask = cfg.x_values >= cfg.linear_min
    return (
        cfg.x_values[mask],
        cfg.y_values[mask],
        cfg.y_errors[mask]
    )


# PERFORMING LINEAR REGRESSION

def perform_standard_linear_regression(x, y):
    slope, intercept, r_value, _, std_err_slope = stats.linregress(x, y)
    n = len(x)
    x_mean = np.mean(x)
    sxx = np.sum((x - x_mean)**2)
    residuals = y - (slope*x + intercept)
    mse = np.sum(residuals**2) / (n-2)
    std_err_intercept = np.sqrt(mse * (1/n + x_mean**2 / sxx))
    return slope, intercept, std_err_slope, std_err_intercept, r_value**2
```

```
def perform_weighted_linear_regression(x, y, y_err):
    weights = 1 / (y_err**2)
    def linear_model(x, a, b): return a*x + b
    popt, pcov = curve_fit(linear_model, x, y, sigma=y_err,
absolute_sigma=True)
    slope, intercept = popt
    std_err_slope, std_err_intercept = np.sqrt(np.diag(pcov))
    # weighted R^2
    y_pred = linear_model(x, slope, intercept)
    ss_res = np.sum(weights*(y - y_pred)**2)
    ss_tot = np.sum(weights*(y - np.average(y, weights=weights))**2)
    r2 = 1 - ss_res/ss_tot
    return slope, intercept, std_err_slope, std_err_intercept, r2

# CALCULATE ONSET VOLTAGE UNCERTAINTY

def calculate_onset_uncertainty(y_threshold, slope, intercept, var_slope,
var_intercept, var_y,
                                x_data=None, y_data=None, y_errors=None,
add_noise=False,
                                noise_level=1.0, num_sims=10000):

    onset_voltages = []

    for _ in range(num_sims):
        if add_noise and x_data is not None and y_data is not None:
            noise = np.random.normal(0, y_errors*noise_level)
            y_noisy = y_data + noise
            a_sample, b_sample, _, _, _ = stats.linregress(x_data, y_noisy)
            y_sample = np.random.normal(y_threshold,
np.sqrt(var_y)*noise_level)
        else:
            a_sample = np.random.normal(slope, np.sqrt(var_slope))
            b_sample = np.random.normal(intercept, np.sqrt(var_intercept))
            y_sample = np.random.normal(y_threshold, np.sqrt(var_y))

        if a_sample <= 0: continue
        onset_voltages.append((y_sample - b_sample)/a_sample)

    onset_voltages = np.array(onset_voltages)
    var_onset_analytical = var_y/slope**2 + ((y_threshold - intercept)**2 *
var_slope)/slope**4 + var_intercept/slope**2
    return {
        'data': onset_voltages,
        'mean': np.mean(onset_voltages),
        'median': np.median(onset_voltages),
        'std': np.std(onset_voltages),
        'p5': np.percentile(onset_voltages, 5),
        'p25': np.percentile(onset_voltages, 25),
        'p75': np.percentile(onset_voltages, 75),
        'p95': np.percentile(onset_voltages, 95),
        'analytical_std': np.sqrt(var_onset_analytical),
        'nominal_onset': (y_threshold - intercept)/slope
    }

#
=============================================================================
```

```python
# PLOTTING
#
================================================================================

def plot_onset_analysis(x_reg, y_reg, y_err_reg, slope, intercept,
results_dict, y_threshold):
    colors = ['#FF6B6B', '#4ECDC4', '#45B7D1', '#96CEB4']
    fig, axes = plt.subplots(3, 3, figsize=(16, 10))
    gs = fig.add_gridspec(3,3,hspace=0.3,wspace=0.3)

    # 1. Overlaid histograms
    ax1 = fig.add_subplot(gs[0,:2])
    for label, res, color in zip(results_dict.keys(), results_dict.values(),
colors):
        ax1.hist(res['data'], bins=50, alpha=0.5, color=color, label=label,
density=True, edgecolor='black')
    ax1.axvline(results_dict[list(results_dict.keys())[0]]['nominal_onset'],
linestyle='--', color='black')
    ax1.set_title('Onset Voltage Distribution')
    ax1.set_xlabel('Voltage (V)')
    ax1.set_ylabel('Probability Density')
    ax1.legend()

    # 2. Box plots
    ax2 = fig.add_subplot(gs[0,2])
    data_list = [res['data'] for res in results_dict.values()]
    ax2.boxplot(data_list, labels=results_dict.keys(), patch_artist=True)
    ax2.set_title('Boxplot Comparison')
    ax2.axhline(results_dict[list(results_dict.keys())[0]]['nominal_onset'],
linestyle='--', color='black')

    # 3. Linear fit with CI
    ax3 = fig.add_subplot(gs[1,:])
    ax3.errorbar(x_reg, y_reg, yerr=y_err_reg, fmt='o', capsize=5,
color='black', ecolor='gray', label='Data')
    x_fit = np.linspace(x_reg.min(), x_reg.max(), 100)
    y_fit = slope*x_fit + intercept
    ax3.plot(x_fit, y_fit, 'r-', label='Linear Fit')
    ax3.axhline(y_threshold, linestyle='--', color='green', label=f'Threshold
y={y_threshold}')
    for res, color in zip(results_dict.values(), colors):
        ax3.axvspan(res['p5'], res['p95'], alpha=0.15, color=color)
    ax3.set_title('Linear Fit with Onset Uncertainty')
    ax3.set_xlabel('Voltage (V)')
    ax3.set_ylabel('Bacteria Count')
    ax3.legend()

    plt.suptitle('Onset Voltage Uncertainty Analysis', fontsize=16,
fontweight='bold')
    plt.show()


# MAIN


if __name__ == "__main__":
    cfg = OnsetConfig()
```

```
    x_reg, y_reg, y_err_reg = select_linear_region(cfg)

    # Weighted regression for slope
    slope, intercept, std_slope, std_intercept, r2 =
perform_weighted_linear_regression(x_reg, y_reg, y_err_reg)
    var_slope = std_slope**2
    var_intercept = std_intercept**2
    var_y = np.mean(y_err_reg)**2

    print(f"Weighted linear regression slope={slope:.4f} ± {std_slope:.4f},
intercept={intercept:.4f} ± {std_intercept:.4f}")

    # Method 1: Sample from parameter distributions
    results_no_noise = calculate_onset_uncertainty(cfg.y_threshold, slope,
intercept, var_slope, var_intercept, var_y,
                                                   add_noise=False,
num_sims=cfg.num_sims)
    print(f"No-noise MC onset voltage: {results_no_noise['mean']:.2f} ±
{results_no_noise['std']:.2f} V")

    # Method 2: Add Gaussian noise at different levels
    results_with_noise = {}
    for nl in cfg.noise_levels:
        label = f"{int(nl*100)}% noise"
        results = calculate_onset_uncertainty(cfg.y_threshold, slope,
intercept, var_slope, var_intercept, var_y,
                                              x_data=x_reg, y_data=y_reg,
y_errors=y_err_reg,
                                              add_noise=True, noise_level=nl,
num_sims=cfg.num_sims)
        results_with_noise[label] = results
        print(f"{label}: mean={results['mean']:.2f} V,
std={results['std']:.2f} V")

    # Include no-noise for comparison
    results_with_noise['No noise'] = results_no_noise

    # Plot results
    plot_onset_analysis(x_reg, y_reg, y_err_reg, slope, intercept,
results_with_noise, cfg.y_threshold)

    # Save results
    save_dict = {'slope': slope, 'intercept': intercept, 'nominal_onset':
results_no_noise['nominal_onset']}
    for label, res in results_with_noise.items():
        key = label.replace(' ', '_').replace('%','pct')
        save_dict[f'{key}_onset_voltages'] = res['data']
        save_dict[f'{key}_mean'] = res['mean']
        save_dict[f'{key}_std'] = res['std']
```

Supplementary Information S2: Separation bacteria of different species and isolates by electrokinetic mobility (EKMr), electrokinetic mobility μ_{EK} and dielectrophoretic mobility μ_{DEP}

Electrokinetic mobility ratio (EKMr) is defined as the ratio of mobilities $\frac{\mu_{EK}}{\mu_{DEP}}$. The lowest potential at which capture occurs (onset potential) is related to the ratio of mobilities, where:

$$\frac{\mu_{EK}}{\mu_{DEP}} = \frac{\nabla|E|^2}{E} \qquad (1)$$

Onset voltage can be determined by fitting a linear regression in a capture voltage regime, as detailed elsewhere [1,2].

Finite-element numerical, performed with the COMSOL software, can calculate EKMr value at the onset potential $\frac{\nabla E^2}{E}$ given the microchannel geometry and capture location.

Electrokinetic mobility μ_{EK} can be determined experimentally as a slope of the linear regression between voltage and particle velocity. The velocity of the particles can be tracked in the region of the linear electric field.

Dielectrophoretic mobility μ_{DEP} can be determined from Equation (1), when both the EKMr and μ_{EK} values are known.

Table S4 and Table S5 summarize the published literature on gradient insular dielectrophoresis (g-iDEP). The sources are listed as experimental, simulation, or literature. Experimental indicates that the value was obtained from imaging/measuring of cell behavior and record of onset voltage, even if the onset voltages were converted to EKMr via COMSOL. Simulation denotes that the value is obtained solely from simulation or calculation. Literature values were obtained from other publications where dielectrophoresis was not necessarily the focus, but the cells were in similar

condition and comparable. These values might be used to convert to other parameters if deemed appropriate.

Table S4: EKMr value of *Staphylococcus epidermidis, Staphylococcus aureus, Listeria moncytogenes and Salmonella.*

**Bacteria isolate**	**Onset voltage (V)**	**EKMr (x 10^9 Vm^{-2})**	**Source**
Staphylococcus epidermidis ATCC 14490, unlabeled	776 ± 29	8.35 ± 0.7	Experimental [7-unpublished data]
Staphylococcus epidermidis ATCC 35984, unlabeled	567 ± 47	11.4 ± 0.4	Experimental [7-unpublished data]
Staphylococcus epidermidis ATCC 35983, labeled	443 ± 59	4.6 ± 0.6	Experimental[3]
Staphylococcus epidermidis ATCC 14490, labeled	881 ± 38	9.2 ± 0.4	Experimental [3]
MRSA, ATCC 43300, labeled	865 ± 71	12.4 ± 1.0	Experimental [4]
MSSA, ATCC 43300, labeled	685 ± 61	9.86 ± 0.89	Experimental [4]
MRSA, ATCC 29213, unlabeled	732 ± 44	10.5 ± 0.6	Experimental [4]
MSSA, ATCC 29213, unlabeled	562 ± 59	8.09 ± 0.85	Experimental [4]
Listeria moncytogenes 1/2a	280 ± 18	2.8 ± 0.2	Experimental [5]
Listeria moncytogenes 1/2b	220 ± 15	2.2 ± 0.2	Experimental [5]
Listeria moncytogenes 4b	220 ± 31	2.2 ± 0.3	Experimental [5]
Salmonella sv. Cubana ATCC 12007	1889 ± 228	27 ± 3	Experimental [2]
Salmonella sv. Poona ATCC BAA-1673	1525 ± 196	22 ± 3	Experimental [2]

Table S5: Electrokinetic mobility μ_{EK} and dielectrophoretic mobility μ_{DEP} of *Staphylococcus epidermidis, Staphylococcus aureus, Listeria moncytogenes and Salmonella.*

**Bacteria isolate**	**μ_{EK} ($m^2V^{-1}s^{-1}$)**	**Source**	**μ_{DEP} ($m^4V^{-2}s^{-1}$)**	**Source**
Staphylococcus epidermidis ATCC 14490, unlabeled	$4.02 \times 10^{-9} \pm 3.00 \times 10^{-10}$	Experimental [7-unpublished data]	$4.81 \times 10^{-19} \pm 5.30 \times 10^{-20}$	Experimental [7-unpublished data]
Staphylococcus epidermidis ATCC 35984, unlabeled	$4.47 \times 10^{-9} \pm 6.10 \times 10^{-10}$	Experimental [7-unpublished data]	$3.92 \times 10^{-19} \pm 5.50 \times 10^{-20}$	Experimental [7-unpublished data]
Staphylococcus epidermidis ATCC 35983, labeled	$4.0 \times 10^{-9} \pm 4.0 \times 10^{-10}$	Literature [6]	$8.7 \times 10^{-19} \pm 8.7 \times 10^{-20}$	Calculated assuming literature
Staphylococcus epidermidis ATCC 14490, labeled	$4.0 \times 10^{-9} \pm 4.0 \times 10^{-10}$	Literature [6]	$4.3 \times 10^{-19} \pm 8.7 \times 10^{-20}$	Calculated assuming literature
MRSA, labeled	$1.25 \times 10^{-8} \pm 5.10 \times 10^{-9}$	Simulation [4]	$1.01 \times 10^{-18} \pm 4.20 \times 10^{-19}$	Experimental [4]
MSSA, labeled	$7.10 \times 10^{-9} \pm 1.60 \times 10^{-9}$	Simulation [4]	$5.59 \times 10^{-19} \pm 1.70 \times 10^{-19}$	Experimental [4]
MRSA, unlabeled	5.51×10^{-9}	Literature [6]	5.25×10^{-19}	Calculated assuming literature
MSSA, unlabeled	5.51×10^{-9}	Literature [6]	6.81×10^{-19}	Calculated assuming literature
Listeria moncytogenes 1/2a	$1.90 \times 10^{-8} \pm 7.00 \times 10^{-10}$	Experimental [5]	$7.0 \times 10^{-18} \pm -3.5 \times 10^{-19}$	Experimental [5]
Listeria moncytogenes 1/2b	$1.70 \times 10^{-8} \pm 7.00 \times 10^{-10}$	Experimental [5]	$7.0 \times 10^{-18} \pm -3.5 \times 10^{-19}$	Experimental [5]
Listeria moncytogenes 4b	$9.20 \times 10^{-9} \pm 3.00 \times 10^{-10}$	Experimental [5]	$4.1 \times 10^{-18} \pm -2.1 \times 10^{-19}$	Experimental [5]
Salmonella sv. Cubana ATCC 12007	$5.00 \times 10^{-8} \pm 5.00 \times 10^{-9}$	Experimental [2]	$1.85 \times 10^{-18} \pm 3.00 \times 10^{-19}$	Experimental [2]
Salmonella sv. Poona ATCC BAA-1673	$6.70 \times 10^{-8} \pm 3.00 \times 10^{-9}$	Experimental [2]	$3.05 \times 10^{-18} \pm 1.80 \times 10^{-19}$	Experimental [2]

Reference

1. Hilton, S.H.; Hayes, M.A. A Mathematical Model of Dielectrophoretic Data to Connect Measurements with Cell Properties. *Anal. Bioanal. Chem.* **2019**, *411*

2. Liu, Y.; Hayes, M.A. Differential Biophysical Behaviors of Closely Related Strains of Salmonella. *Front. Microbiol.* **2020**, *11*, 524478

3. Jones, P. V.; Huey, S.; Davis, P.; Yanashima, R.; McLemore, R.; McLaren, A.; Hayes, M.A. Biophysical Separation of Staphylococcus Epidermidis Strains Based on Antibiotic Resistance. *Analyst* **2015**, *140*, 5152–5161

4. Hilton, S.H.; Crowther, C. V.; McLaren, A.; Smithers, J.P.; Hayes, M.A. Biophysical Differentiation of Susceptibility and Chemical Differences in: Staphylococcus Aureus. *Analyst* **2020**, *145*, 2904–2914

5. Crowther, C. V.; Hilton, S.H.; Kemp, L.K.; Hayes, M.A. Isolation and Identification of Listeria Monocytogenes Utilizing DC Insulator-Based Dielectrophoresis. *Anal. Chim. Acta* **2019**, *1068*, 41–51

6. Bayer, M.E.; Sloyer, J.L. The Electrophoretic Mobility of Gram-Negative and Gram-Positive Bacteria: An Electrokinetic Analysis. *J. Gen. Microbiol.* **1990**, *136*, 867–874

7. Nguyen, H.; Rasel, A.K.M.F.K.; McLaren, A.; Hayes, M.A. *Intrinsic Precision for Electrophysical Characterization of Minimized Biological Variance Populations of Bacteria using Dielectrophoresis*. **Biomicrofluidics**, manuscript submitted.
